# The relationship between the ‘‘Fujisawa point’’ and anatomical femorotibial angle following simulated open wedge high tibial osteotomy

**DOI:** 10.1186/s12891-022-05734-7

**Published:** 2022-08-15

**Authors:** Hideo Kobayashi, Suguru Saito, Yasushi Akamatsu, Ken Kumagai, Shuntaro Nejima, Yutaka Inaba

**Affiliations:** 1 Department of Orthopaedic Surgery, Yokohama City University School of Medicine, 3-9 Fukuura, Kanazawa, Yokohama, Kanagawa 236-0004 Japan; 2 Department of Orthopaedic Surgery, Yokohama Hodogaya Central Hospital, Yokohama, Japan; 3 Department of Orthopaedic Surgery, International University of Health and Welfare Atami Hospital, Atami, Japan; 4 Department of Joint Surgery, Fureai Yokohama Hospital, Yokohama, Japan

**Keywords:** Open wedge high tibial osteotomy (OWHTO), Fujisawa point, Femorotibial angle (FTA), Hip-knee-ankle angle (HKA), Neck shaft angle

## Abstract

**Background:**

We evaluated the relationship between the weight-bearing line (WBL) ratio and anatomical femorotibial angle (FTA) by simulated open wedge high tibial osteotomy (OWHTO). This study evaluated the correlation between the ‘‘Fujisawa point’’ and FTA, and identified factors which caused deviations between the two measurement methods. We hypothesized that the Fujisawa point corresponded with 170° of the FTA.

**Methods:**

Preoperative antero-posterior full-length lower limb radiographs of 82 patients were obtained for the OWHTO to place the WBL ratio at a target of 62.5% of the width of the tibial plateau (Fujisawa point). The coronal alignment was measured pre- and post-planning. The patients were divided into two groups by the post-planning FTA: a correspondence group (168.5°≦FTA≦171.5°) and a non-correspondence group (FTA < 168.5°, 171.5° < FTA). The relationship between the Fujisawa point and the FTA was analyzed with multivariate regression analysis.

**Results:**

The post-planning FTA was 169.8 ± 1.1° and within 170 ± 1.5° in 69 cases (84.1%) when the WBL ratio was 62.5%. The neck shaft angle was 128.1 ± 5.2° in the correspondence group, and 122.3 ± 6.3° in the non-correspondence group. The multivariate linear regression analysis revealed that the neck shaft angle was the only factor that predicted the correspondence of the Fujisawa point with the FTA at 170° (*p* = 0.006, odd 1.28).

**Conclusions:**

The post-planning FTA converged at 170° when the WBL ratio passed through the Fujisawa point and the neck shaft angle was the only predictor.

**Supplementary Information:**

The online version contains supplementary material available at 10.1186/s12891-022-05734-7.

## Background

Postoperative lower limb alignment is the most important factor that influences clinical outcomes and longevity for medial open wedge high tibial osteotomy (OWHTO) [1, 2]. Careful and precise preoperative planning is mandatory to avoid both under-correction and over-correction. The weight-bearing line (WBL) ratio, anatomical femorotibial angle (FTA), or hip-knee-ankle angle (HKA) is mainly referred for preoperative planning and postoperative radiological evaluation [3–5]. The FTA, which is the lateral angle between the femoral anatomical axis and the tibial anatomical axis, has already been established as a factor that can evaluate knee alignment including osteotomies around the knee and is originally measured using standard antero-posterior knee radiographs, which are easier to handle than the antero-posterior full-length lower limb radiographs [3, 4, 6]. Coventry et al. has reported that a post-operative FTA more than 172° resulted in a significantly higher failure rate in proximal tibial osteotomy [6]. In osteotomies around the knee, many authors have defined the preoperative planning angle and postoperative optimal alignment as a FTA of 170° [3, 4] and reported satisfactory clinical outcomes. However, the FTA may fail to reflect correct lower limb alignment because it does not take into consideration the influence of lower leg length and femoral/tibial bowing. On the other hand, the WBL ratio or HKA measured on full-length lower limb radiographs is considered the gold standard, as it allows for reliable and accurate measurement of the whole lower extremity without the influence of lower leg length and femoral/tibial bowing. The WBL ratio or HKA has been used in the navigation system during operation [7]. Postoperative optimal alignment is defined to be between 3° to 6° of the mechanical valgus, or between 60 and 70% of the WBL ratio [8]. Fujisawa et al. reported that the corrected axis should run through a target zone between 30 and 40% lateral to the midpoint of the knee for optimal results [9]. Based on this conclusion, 62.5% of the WBL ratio, also recognised as the ‘‘Fujisawa point’’ is widely accepted as the target postoperative alignment [5, 10, 11]. A few studies report reciprocal relationships between the measurement methods [3, 4, 12]. The optimal WBL ratio is reported to be 62% when the anatomical FTA is 170° [13]. The differences in individual anatomical bone morphology, such as femoral bowing, tibial bowing, lower leg length, and neck shaft angle, may cause deviations in the results among different methods of measurement such as the WBL ratio, FTA, and HKA.

The aims of the study were (i) to investigate the relationship between the WBL ratio and the FTA, which in essence aims to establish a correlation between the Fujisawa point and the FTA, and (ii) to identify factors which result in deviations between the two measurement methods after simulated OWHTO. We hypothesized that the Fujisawa point converged with the FTA at 170°, and some factors would cause deviations between the Fujisawa point and the FTA at 170°.

## Methods

### Patients

In total, 82 knees on 77 patients with medial knee osteoarthritis undergoing OWHTO between April 2012 and April 2016 were included in this study. Our Institutional Review Board approved the study, and all the patients provided informed consent prior to participating. The patients included 38 men and 39 women with a mean age of 69.2 years (range: 40–86 years). Table 1 represents the clinical data of patients.

**Table 1:** Demographic characteristics of the enrolled patients

	Series (*n* = 82)	Correspondece (*n* = 69)	Non-correspondence (*n* = 13)	*P* value
		168.5° ≦ FTA ≦1 71.5	FTA < 168.5°, 171.5° < FTA	
Age (years)	69.2	69.5	67.4	0.32
	67.3—71.0	67.4—71.5	62.6—72.2	
Body height (cm)	161.3	160.7	164.6	0.29
	158.9—163.7	158.1—163.3	157.6—171.5	
Body weight (kg)	67.3	66.5	73.4	0.08
	63.4—71.3	61.9—71.1	65.4—81.4	
Body mass index (kg/m^2^)	25.5	25.4	27.0	0.08
	24.5—26.5	24.2—26.5	24.7—29.3	

*FTA* Femorotibial angle

### Radiographic evaluation

**Fig. 1:**
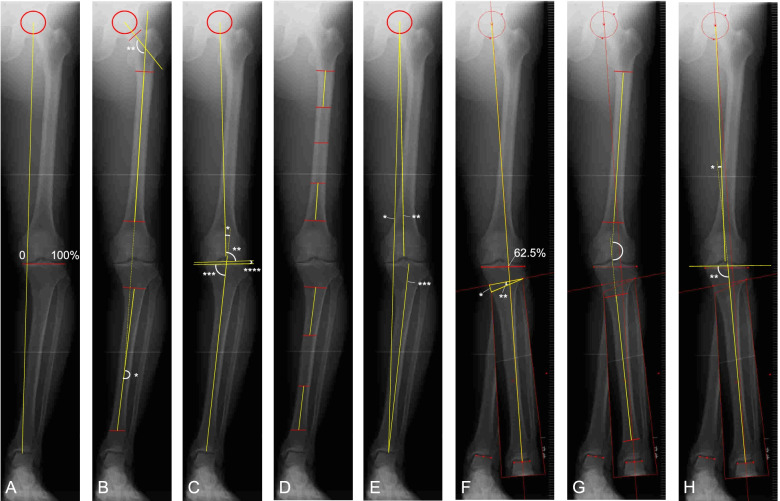
Preoperative standing anteroposterior radiographs of the full-length lower limb. **A-E** pre-simulation, **F–H** post-simulation. For simulation, the weight bearing line (WBL) was drawn from the center of the femoral head to 62.5% of the width of the tibial plateau (Fujisawa point). The distal tibia was rotated till the WBL passed through the 62.5% coordinate. **A** WBL ratio: The WBL was drawn from the center of the femoral head to the center of the dome of the talus. The WBL ratio was defined as a ratio of the tibial width which is measured from the medial side to the lateral side. **B** Pre-simulation (*) / **G** post-simulation femorotibial angle (FTA): FTA was defined as a lateral angle between the femoral anatomical axis and the tibial anatomical axis. **B** Neck shaft angle (**) was defined as an angle between the midline drawn in the femoral neck and the femoral anatomical axis. **C** Pre-simulation (*) / **H** post-simulation (*) hip-knee-ankle axis (HKA) angle: HKA was formed by the mechanical axes of the femur and tibia. **C** Pre-simulation mechanical lateral distal femoral angle (mLDFA) (**) was defined as the lateral angle between the mechanical femoral axis and an articular tangential line of the distal femur. **C** Pre-simulation (***) / **H** post-simulation (**) mechanical medial proximal tibial angle (mMPTA) was defined as the medial angle between the mechanical tibial axis and an articular tangential line of the proximal tibia. **C** Pre-simulation joint line convergence angle (JLCA) (****) was defined as the angle between an articular tangential line of the distal femur and an articular tangential line of the proximal tibia. **D** Coronal femoral/tibial bowing: the femoral diaphysis was divided into four equal parts, and the midpoint of the endosteal intramedullary canal was depicted in each quarter. The angulation between midlines drawn in the proximal and distal quarters of the femoral diaphysis was measured. The tibial diaphysis was divided into three equal parts, and the angulation between midlines drawn in the proximal and distal thirds of the tibial diaphysis was measured. **E** Pre-simulation lower leg length (*) was defined as the distance between the center of the femoral head and the center of the dome of the talus. **E** Femoral leg length (**) was defined as the distance between the center of the femoral head and the most proximal point of the femoral notch. **E** Pre-simulation tibial leg length (***) was defined as the distance between the midpoint of the tibial spines and the center of the dome of the talus. **F** Opening gap (*) and opening angle (**) were measured

Antero-posterior full-length lower limb radiographs were obtained preoperatively with patients in the one-leg standing position. The radiographs were analyzed using the Fujifilm OP-A software (Fujifilm, Co., Ltd, Tokyo, Japan), and measurements of various parameters and planning of OWHTO were performed (Fig. 1).

### Planning method

The osteotomy line was set from the medial side, 35 mm distal to the medial tibiofemoral joint line, to the proximal tibiofibular joint. The WBL refers to a line drawn from the center of the femoral head to the center of the dome of the talus and the WBL ratio is defined as a ratio of the tibial width which was measured from the medial side to the lateral side (Fig. 1A). The center of the femoral head was identified by fitting a circle on the femoral head. Following Dugdale and Noyes’ planning method [14], a post-planning WBL was drawn from the center of the femoral head to 62.5% of the width of the tibial plateau. The distal tibia was rotated around the hinge point till the WBL passed through the 62.5% coordinate (Fig. 1F-H). In order to identify factors that caused deviations between the WBL ratio and the FTA, the patients were divided into two groups by the post-planning FTA: a correspondence group (168.5°≦FTA≦171.5°) and a non-correspondence group (FTA < 168.5°, 171.5° < FTA). In our clinical study of OWHTO related to joint line convergence angle using a navigation system, 96 knees were categorized in the acceptable (absolute navigation correction loss (NCL) value≦1.5°) and outlier groups (absolute NCL value > 1.5°) [15]. Absolute value of 1.5° was used to divide into two groups in the present study. No significant differences in patient characteristics were found between the two groups; the clinical data are provided in Table 1.

### Measurements

The following angles and distances were measured: pre-planning WBL ratio (Fig. 1A), pre-planning / post-planning FTA (Fig. 1B, G), pre-planning / post-planning HKA (Fig. 1C, H), pre-planning mechanical lateral distal femoral angle (mLDFA, Fig. 1C), pre-planning / post-planning mechanical medial proximal tibial angle (mMPTA, Fig. 1C, H), pre-planning joint line convergence angle (JLCA, Fig. 1C), and pre-planning joint line obliquity (JLO) angle, coronal bowing of both femur and tibia (Fig. 1D) [16], pre-planning lower leg lengths (Fig. 1E), pre-planning femoral leg length (Fig. 1E), pre-planning tibial leg lengths (Fig. 1E), neck shaft angle (Fig. 1B), opening gap (Fig. 1F), and opening angle (Fig. 1F). The FTA was measured as the lateral angle between the femoral anatomical axis and the tibial anatomical axis (Fig. 1B, G). The mechanical femoral axis was defined as the line joining the center of the femoral head and the center of the knee. The mechanical tibial axis was defined as the line joining the center of the knee and the center of the dome of the talus. The center of the knee was defined as the midpoint of the tibial spines. The HKA was measured as the angle formed by the mechanical axis of the femur and the mechanical axis of the tibia (Fig. 1C, H). mLDFA was defined as the lateral angle between the mechanical femoral axis and an articular tangential line of the distal femur (Fig. 1C). mMPTA was defined as the medial angle between the mechanical tibial axis and an articular tangential line of the proximal tibia (Fig. 1C, H). JLCA was defined as the angle between an articular tangential line of the distal femur and an articular tangential line of the proximal tibia (Fig. 1C). JLO was defined as the angle between a line parallel to the ground and an articular tangential line of the proximal tibia. Lower leg length was defined as the distance between the center of the femoral head and the center of the dome of the talus (Fig. 1E). Femoral leg length was defined as the distance between the center of the femoral head and the most proximal point of the femoral notch (Fig. 1E). Tibial leg length was defined as the distance between the midpoint of the tibial spines and the center of the dome of the talus (Fig. 1E).

### Statistical analysis

All data were analyzed using SPSS for Windows (version 26). The data were expressed as means with 95% confidence intervals (CIs). The results between the correspondence group and the non-correspondence group were compared using the Mann Whitney test. Following the univariate analysis, the correlations between variables including body height, body weight, pre-planning FTA, WBL ratio, pre-planning / post-planning HKA, neck shaft angle, tibial bowing, opening gap, and opening angle, were analyzed with multivariate regression analysis to evaluate the correspondence between the Fujisawa point and the FTA at 170°. Associations among pre-planning FTA, pre-planning HKA, and pre-planning WBL ratio and between post-planning FTA and post-planning HKA were evaluated using the Pearson’s correlation coefficient. *P* values less than 0.05 were considered statistically significant.

## Results

**Fig. 2:**
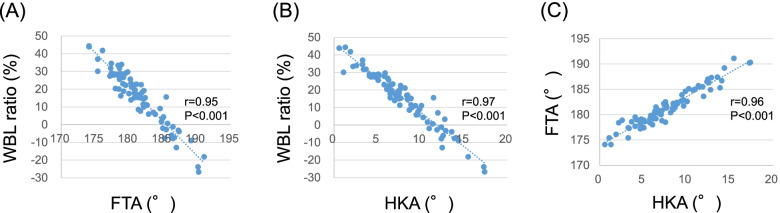
A correlation was observed **A** between pre-simulation FTA and pre-simulation WBL ratio, **B** between pre-simulation HKA and pre-simulation WBL ratio, and **C** between pre-simulation FTA and pre-simulation HKA

The pre-planning WBL ratio was 15.6%. The pre-planning FTA was 181.5 ± 3.7°, and the post-planning FTA was 169.8 ± 1.1° (Table 2). The pre-planning HKA was 7.9 ± 3.7°, and the post-planning HKA was -2.9 ± 0.6° (Table 2). There were significant correlations between pre-planning FTA and pre-planning WBL ratio (*r* = 0.95, *P* < 0.001), pre-planning HKA and pre-planning WBL ration (*r* = 0.97, *P* < 0.001), and pre-planning FTA and pre-planning HKA (*r* = 0.96, *P* < 0.001) (Fig. 2).

**Table 2:** Angular and linear measurements of the correspondence group and the non-correspondence group

	Series (*n* = 82)	Correspondece (*n* = 69)	Non-correspondence (*n* = 13)	*P* value
		168.5° ≦ FTA ≦ 171.5	FTA < 168.5°, 171.5° < FTA	
**Pre-planning measurements**				
FTA (°)	181.5	181.3	182.1	0.82
	180.7—182.3	180.4—182.2	179.5—184.8	
WBL ratio (%)	15.6	17.2	9.1	0.12
	12.3—18.9	13.7—20.7	-1.1—19.4	
lower leg length (mm)	767.0	766.5	771.3	0.63
	754.4—779.7	752.3—780.6	735.6—807.0	
femoral leg length (mm)	416.0	416.0	416.8	0.95
	409.5—422.5	408.7—423.2	397.8—435.8	
tibial leg length (mm)	345.8	345.1	345.0	0.42
	339.5—352.1	338.1—352.2	333.0—367.0	
neck shaft angle	127.2	128.1	122.3	0.003
	125.9—128.5	126.8—129.4	118.5—126.1	
HKA (°)	7.9	7.5	9.3	0.21
	7.1—8.7	6.7—8.4	6.7—11.8	
mLDFA (°)	89.8	89.6	90.6	0.30
	89.1—90.4	88.9—90.3	88.5—92.7	
mMPTA (°)	84.1	84.2	83.3	0.10
	83.5—84.7	83.7—84.8	81.6—85.0	
JLCA (°)	2.5	2.5	2.8	0.57
	2.2—2.9	2.2—2.9	1.9—3.7	
JLO (°)	2.5	2.6	1.8	0.37
	2.1—3.0	2.1—3.1	0.8—2.9	
femoral bowing (°)	0.4	0.2	0.9	0.88
	-0.5—1.2	-0.6—1.1	-2.1—3.9	
tibial bowing (°)	0.4	0.3	0.8	0.49
	-0.1—0.8	-0.2—0.7	-0.9—2.4	
**Post-planning measurements**				
FTA (°)	169.8	169.9	168.9	0.002
	169.5—170.0	169.8—170.1	167.7—170.0	
opening gap (mm)	13.9	13.4	16.0	0.18
	13.0—14.8	12.5—14.4	12.7—19.3	
opening angle (°)	12.0	11.6	13.5	0.18
	11.2—12.8	10.8 -12.5	10.9—16.1	
HKA (°)	-2.9	-2.9	-2.7	0.64
	-3.0—-2.7	-3.0—-2.7	-3.3—-2.1	
mMPTA (°)	95.0	94.9	95.5	0.30
	94.4—95.5	94.3—95.4	93.2—97.7	

*FTA* Femorotibial angle, *WBL* Weight bearing line, *HKA* Hip-knee-ankle angle

*mLDFA* Mechanical lateral distal angle, *mMPTA* Mechanical medial proximal tibial angle, *JLCA* Joint line convergence angle, *JLO* Joint line obliquity

**Fig. 3:**
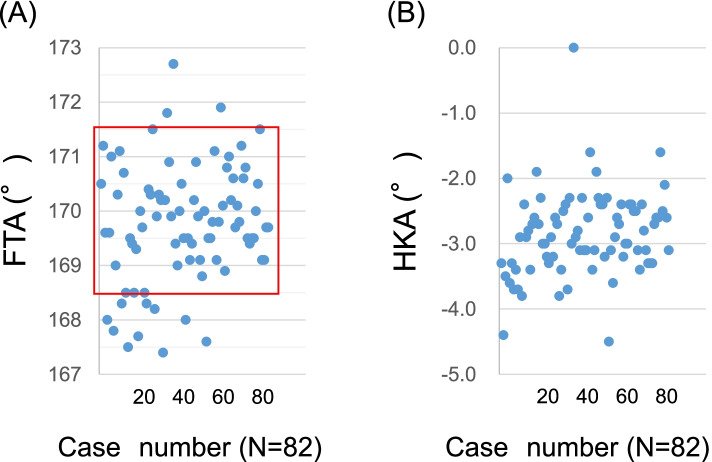
**A** The scatter diagram of the post-simulation FTA when the WBL passed through Fujisawa point in each case. The post-simulation FTA was 169.8 ± 1.1°. The FTA was within 170 ± 1.5° in 69 cases (84.1%). **B** The scatter diagram of the post-simulation HKA when the WBL passed through Fujisawa point in each case

The post-planning FTA was within 170 ± 1.5° in 69 cases (84.1%) when the WBL ratio was 62.5% (Fujisawa point, Fig. 3). There was a difference in the neck shaft angle between the correspondence group (128.1 ± 5.2°) and the non-correspondence group (122.3 ± 6.3°) (*P* = 0.003, Table 2). The multivariate regression analysis revealed that the neck shaft angle was the only factor that predicted the correspondence of the Fujisawa point and the FTA at 170° (Odds ratio 1.28, *P* = 0.006). There were no significant correlations between post-planning FTA and post-planning HKA.

## Discussion

The important findings of the present study were: (i) The Fujisawa point converged with the FTA at approximately 170°. (ii) The neck shaft angle was the only factor that predicted the correspondence of the Fujisawa point and the FTA at 170°.

In the clinical and radiographic evaluation of 57 cases that underwent the OWHTO aiming at a postoperative FTA of 170°, the postoperative FTA and the WBL ratio were 169.6° ± 2.3° and 62.9% ± 12.5%, respectively, and a significant correlation was evident between them after the OWHTO (*R*^*2*^ = 0.385, *P* < 0.01) [13]. In a previous prospective multicenter study of 118 cases that underwent an OWHTO aiming at a postoperative FTA of 170° or 62.5% of the WBL ratio, the FTA and WBL ratio changed significantly from 179.3° (95% CIs: 178.7°-179.9°) to 169.8° (169.2°-170.5°) and from 23.1% (20.7–25.5%) to 62.4% (59.0–65.8%), respectively [3]. Similarly, many authors have planned and achieved postoperative alignment at an FTA of 170° in osteotomies around the knee for patients with knee osteoarthritis or osteonecrosis, and satisfactory clinical outcomes have been reported [4, 12, 17, 18]. The relationship between the postoperative FTA and the WBL ratio is similar to that revealed in our study and these previous studies and our study support a FTA of 170° as the postoperative target angle in OWHTO [3, 4, 12, 13, 17, 18]. Yin Y et al. [5] determined the relationship between the Fujisawa point and the postoperative HKA and the anatomical factors, such as preoperative HKA, femoral length, tibial length, and tibial plateau width, that influence this relationship. In 116 simulated OWHTO, the HKA through the Fujisawa point was 2.4° (2.1–2.7°) and the preoperative HKA was a significant contributor to the postoperative valgus angle in the multivariate regression analysis. The authors noted that a patient with more severe medial knee osteoarthritis tended to have a lower valgus angle after OWHTO according to the Fujisawa point. In our study, the multivariate regression analysis revealed that the neck shaft angle was the only predictor in the correspondence and the non-correspondence groups.

The neck shaft angle generally falls within a range of 120° and 140°. A global neck shaft angle database comprising of over 8000 femora representing 100 human groups reported that the mean neck shaft angle for modern humans is between 127° and 129° in Asia [19]. In the study of the morphological change of 1538 knees in progressing varus knee osteoarthritis, Lu et al. have shown that the neck shaft angle decreased significantly by age in females (the neck shaft angle of each age group; < 40, 40–60, and > 60 was 134.92 ± 3.85°, 128.35 ± 6.37°, and 128.84 ± 6.18°, respectively) and the femoral bowing angle positively influenced the neck shaft angle as femoral bowing lead to corresponding changes in both ends of the femur with the progression of knee osteoarthritis [20]. Similarly, the neck shaft angle is reported to have a significant negative correlation with age [19, 21]. Regarding the relationship between the neck shaft angle and the FTA, the neck shaft angle was smaller in the non-correspondence group compared with the correspondence group in our study and a previously reported angle [19]. Although there are no reports describing the relationship between the neck shaft angle and lower limb alignment, the decrease of the neck shaft angle theoretically results in the increase of the horizontal distance between the femoral head and the proximal femur, which might cause a discrepancy between the FTA and the WBL ratio. On the other hand, femoral bowing and tibial bowing did not influence the results in two groups. Coronal bowing of the femur and tibia were an average of 3.0° (-7.4° to 10.9°) and 0.4° (-4.1° to 4.6°) respectively, in seventy bilateral TKAs in 35 patients with knee osteoarthritis [16]. The femoral bowing angles were 2.40 ± 2.63° (-8°—14°) in females and 1.82 ± 2.26° (-5°—9°) in males in a previous study [20]. The bowing angle may be too small (femoral bowing 0.4°, tibial bowing 0.4°) to cause a discrepancy between the two groups in our study.

Optimal postoperative lower leg alignment including MAD ratio and HKA remain controversial. Target postoperative HKA is reported to be 1° to 3° valgus [22], 3° to 5° valgus [23], or 3° to 6° valgus [8]. Especially, 3° to 6° valgus of HKA is recognized as the ‘‘ideal correction’’ of the postoperative alignment. In the systematic review, 2341 patients across 39 articles including 50 cohorts were investigated to determine whether coronal angular corrections correlate with patient reported outcomes after HTO [24]. The HKA angle was corrected from 7.1 ± 1.7° (4.1° to 10.6°) varus to 2.3 ± 1.7° valgus (-1.4° to + 6.5°), i.e. patients did not achieve the ‘‘ideal correction’’ of 3° to 6° valgus postoperatively. The authors concluded the fact of clinically and statistically important improvements in patient-reported outcome measure scores suggests that the ‘‘ideal correction’’ may be more flexible than 3° to 6° valgus. When the HKA was set at 4.5° valgus (midpoint of 3° to 6°), the MAD ratio was 71.93% (67–78%) in the previous study [5]. In our study, the post-planning HKA was 2.9 ± 0.6° valgus when the WBL ratio was 62.5%, which is thought to be the optimal postoperative alignment.

This study has several limitations. First, the preoperative radiograph was used for pre-planning and post-planning measurements, and the actual postoperative radiograph was not evaluated. Various factors, such as tibial rotation by osteotomy, change of JLCA, and position of radiograph, such as flexion or rotation, affect the postoperative radiograph. Especially, it is difficult to predict postoperative JLCA and various studies have reported for the prediction [15, 25]. The change in JLCA is related to the postoperative limb alignment such as correction error and overcorrection [26, 27]. Therefore, further study to compare planned measurements with actual postoperative measurements might be a key to resolve the coronal correction discrepancy. However, despite this, we could purely evaluate the influence of the osteotomized gap. Second, some authors recommend that both-leg standing radiographs should be used routinely for preoperative evaluation [28]. On the other hand, no differences have been observed between the one-leg and both-legs views in cases of knee osteoarthritis [29]. One-leg weight bearing radiographs of the full-length lower limb were measured in our study, because the method enables the joint line of the knee to be parallel to ground if it is normal and is used in many clinical studies related to high tibial osteotomy [15, 25, 30, 31].

Our results, in terms of clinical significance, show that it is appropriate to use 170° as an accurate reference point of the FTA as a target angle for OWHTO in case of the absence of hip deformity. The proximal femoral deformity should be taken into consideration in cases where the knee radiograph is used for the preoperative planning or postoperative evaluation. The WBL ratio should be used in patients with a small neck shaft angle. The smaller neck shaft angle has an impact on the correspondence of the Fujisawa point with the FTA at 170°. Further study should be focused on the influence of the larger neck shaft angle.

## Conclusion

The Fujisawa point converged with the FTA at 170°. The neck shaft angle was the sole predictor of the correspondence of the Fujisawa point with the FTA at 170° as the lower neck shaft angle is associated with a lower correspondence between the Fujisawa pint and the FTA at 170°.

## Supplementary Information

**Additional file 1.**

## Data Availability

All data generated or analysed during this study are included in the supplementary table.
